# Responsible innovation and ethical corporate behavior in the Asian fashion industry: A systematic literature review and avenues ahead

**DOI:** 10.1007/s10490-022-09844-7

**Published:** 2022-08-24

**Authors:** Assunta Di Vaio, Rohail Hassan, Gabriella D’Amore, Riccardo Tiscini

**Affiliations:** 1 Department of Law, University of Naples “Parthenope”, G. Parisi, no. 13, 80132 Naples, Italy; 2 Othman Yeop Abdullah Graduate School of Business (OYAGSB), Universiti Utara Malaysia (UUM), 50300 Kuala Lumpur, Malaysia; 3 Mercatorum University of Rome, Piazza Mattei, no. 10, 00186 Roma, Italy

**Keywords:** Corporate social responsibility (CSR), Technological innovation, Innovative business models, Sustainable development goals (SDGs), Bibliometric analysis

## Abstract

Fashion firms have transferred their manufacturing processes to Asia, seeking minimum labor costs, supported by the academic literature’s proposals for alternative supply chain configurations to maximize profits. Fashion industry has undergone public analysis, facing demands for greater transparency about environmental and social sustainability. The growing public awareness of sustainability issues has led firms to declare their commitment to sustainable resources, but few changes have been registered. United Nations Economic Commission for Europe listed technological innovation as a key tool for making the fashion industry transparent and traceable regarding sustainability and circularity. The research and responsible innovation framework indicate appropriate ways to manage innovation from a responsible and ethical perspective, according to ethical corporate behaviour (ECB), particularly in the industries characterized by productive phases processed in more countries, such as the fashion industry. However, the linkages between responsible innovation, ECB towards innovative and sustainable business models, and their conceptualization, are still unclear in the fashion industry, achieving the goals included in the UN 2030 Agenda. This study draws on bibliometric analysis and systematic review of the literature on 114 articles published between 1990 and 2021 allows to identify the above issues in the research domains, and outline the evolutionary trajectories, as well as to explore the literary corpus about responsible innovation (RI) in the ethical corporate behaviour (ECB) of the fashion industry and its Asian suppliers. The results highlight that fashion brands strive to develop RI and ECB along their supply chain. Still, the misalignment of corporate ethics and cultural values represents a significant obstacle to the adoption of business models, especially to achieve the goals of UN 2030 Agenda. To the best of our knowledge, this is the first study that discusses RI as enabling driver in the ECB for fashion companies also defining a future research agenda including RI, ECB, iSBMs towards SDGs.

Over the last five decades, the fashion industry has been increasingly analyzed in the sustainability framework, particularly for the environmental impacts and employees’ working conditions (Garcia-Torres et al., 2021). This industry involves several production steps and suppliers, often located in low-income countries, such as Bangladesh, Vietnam, and India, where employees' health and working conditions are not strictly regulated and monitored by the countries’ governments (Pedersen & Andersen, 2015). Since 2013 the Rana Plaza collapse in Savar, Bangladesh, was considered the worst accident that has ever happened in a textile factory, especially for the negligence of the textile factories owners by not securing workers' lives. This event has been a flywheel in need of transparency in the fashion manufacturing supply chain (Henninger et al., 2016), but the good practices to improve working conditions in the fashion industry remain not identifiable (Bhandari et al., 2022), as well as the initiatives for reducing environmental pollution (Rinaldi et al., 2022). Hence, the fashion industry still ranks second after the oil industry regarding waste production (Niinimäki et al., 2020; Sadowski et al., 2021). Recent events, e.g., the COVID-19 pandemic, have unearthed the need to protect the environment and human health through sustainable and responsible production and consumption models fitting the sustainable development goals (SDGs) included in the United Nations (UN) 2030 Agenda establishing an action program for people, the planet, prosperity, and peace (de Paula Arruda Filho, 2017). To achieve sustainability management in the fashion supply chain and “traceability” and “transparency” tools (Garcia-Torres et al., 2021), scholars (Bhandari et al., 2022; Pal et al., 2019) and practitioners (McKinsey & Company, 2020) are seeking innovative solutions to meet UN 2030 Agenda. Likewise, traceability and transparency issues represent the enabling drivers for sustainability and circularity in the garment and footwear industry, as also enabling pillars for a responsible business behaviour from consumers, governments, and civil society (United Nations Economic Commission for Europe, UNECE, 2021).

Some scholars (Di Vaio et al., 2020; Vacchi et al., 2021) have glimpsed technological innovation as the key pillar in achieving sustainable business models (SBMs), as well as the UNECE (2022) defined the “advanced technologies” as all tools to enhance traceability and transparency towards the circular and sustainable economy of fashion value chains. However, technological innovation could be ineffective without ethics and responsibility in the corporate governance of business organizations (Blok & Lemmens, 2015; Von Schomberg, 2013). The European Commission introduced the concept of research and responsible innovation (RI) has been introduced in 2002 by European Commission through the Program for Research and Technological Development (Jacob et al., 2013). A set of actions aimed to “ensure desirable effects of technology and capture a high level of responsibility in R&I initiatives” (Yaghmaei, 2018, p. 216). Under this lens, some scholars have analyzed the innovation processes to understand the proper ways to manage innovation, fitting the responsibility code of business organizations (Brand & Blok, 2019; Hartley et al., 2017). Hence, corporate behaviour should include a proactive approach to ethical and social issues linked to the technological innovations in the supply chain to handle in the corporate architecture before the adoption in the operational processes (Ribeiro et al., 2016). Likewise, the RI concept had already been discussed by Snihur and Zott (2013): to get SBMs, it is required RI should be included in the ethical code of corporate organizations, thus in the ethical corporate behaviour (ECB). It should also be relevant to have ethics policy to ensure that sustainable economic growth must not occur at the expense of living conditions for workers in the fashion industry, especially in developing countries and emerging economies. Indeed, the lack of ethical policies in a business organization (Chan et al., 2020) or “eco-literate and skilled employees” and “insufficient commitment from top management” (Bhandari et al., 2022) hinder the transition towards innovative SBMs (Rathinamoorthy, 2019). These represent barriers to a transparent, traceable, and sustainable supply chain enforcing the idea that corporate social responsibility (CSR) cannot be supported mainly by seeking social legitimacy and reputation (Snihur & Zott, 2013).

On the other hand, introducing technological innovation issues as enabling key for the sustainability of business in the fashion supply chain highlights the linkage with open innovation (Blok & Lemmens, 2015; Gassman & Enkel, 2004; Long & Blok, 2018) and the engagement of stakeholders (Jarmai & Vogel-Pöschl, 2020). Hence, RI has been considered a leading solution to manage innovation development toward global sustainability challenges meeting SDGs (Lubberink et al., 2017; Ribeiro et al., 2016; Silva et al., 2019). In more detail, RI appears as a governance framework in which “societal actors and innovators become mutually responsive to each other with a view to the (ethical) acceptability, sustainability and societal desirability of the innovation process and its marketable products” (Von Schomberg, 2013).

This perspective makes RI particularly suitable for leading the fashion industry toward the UN 2030 Agenda. Nevertheless, most research about RI has been developed from a policy or socio-ethical perspective, while there are few studies about RI adoption in a specific industry (Blok, 2019; Blok & Lemmens, 2015; Lubberink et al., 2017). The linkages between the issues introduced above, as well as their conceptualization, are still unclear in the fashion industry, especially "how" and "when" and "what" RI governance framework enables to move from the traditional fashion industry to an “ethical and environmentally sustainable” industry. Thence, RI and ECB towards innovative SBMs (iSBMs).

This study seeks to fill this gap through a systematic literature review (SLR) (Bonilla et al., 2015; Donthu et al., 2021) analyzing of the literary corpus about RI in enabling ECB in the fashion industry. This SLR allows to systematize and classify the main contributions in the field, highlighting unexplored characteristics and suggesting a future research agenda (Lim et al., 2022; Martins et al., 2019; Paul et al., 2021; Snyder, 2019; Webster & Watson, 2002; Xiao & Watson, 2019). It provides a quantitative overview of the academic literature that constitutes the field. Moreover, through the qualitative analysis, this study seeks to clarify the role played by technological innovation and the values of sustainability and ethics in the governance of iSBMs in achieving the SDGs outlined in the UN 2030 Agenda.

In line with the literature about the research questions (RQs)(Alvesson & Sandberg, 2011; Sutton & Staw, 1995; Whetten, 1989), below were developed the RQs:

- RQ1: What is the role of RI in enabling ECB in the fashion industry?
- RQ2: How can technological innovation help Asian fashion companies to overcome the trade-off between profit and social responsibility?
- RQ3: How can technological innovation allow Asian fashion companies to adopt innovative business models for the SDGs?

With the intention of contributing to the intersection of open innovation theory, stakeholder theory, and legitimacy theory, this study carries a bibliometric analysis of data composed of documents collected from 114 articles published in the English language between 1990 and 2021 via detailed searches in the Scopus database and Google Scholar (GS), followed by descriptive, bibliometric and network analysis using the tools such as MS Excel (2019), Voyant Tools and VOSviewer. Microsoft Excel is powerful data visualization and analysis software (Raubenheimer, 2017). This analysis provides a clear representation of networks among scholars allowing them to identify “how” the knowledge ranks in the research domains as well as to plot the main evolutionary trajectories (Krishen et al., 2021). In addition, although the SLR identifies and discusses the conceptual and methodological issues in previous studies to systematize patterns and knowledge (Haddoud et al., 2021).

The results highlight that CSR and legitimacy theory are the main approaches to investigate these issues, and qualitative methods are preferred to quantitative analyses.

This study provides evidence of a gap in the theoretical approach used by researchers to analyze the sustainability issues in the fashion industry, disregarding the potentialities of RI to drive the change along the supply chain. This study discusses how RI can improve fashion companies’ sustainability by identifying the antecedents that allow Asian companies to adopt ECB. Besides, this study highlights how open innovation theory can support the RI theoretical framework in becoming substantial by developing innovative, sustainable, and ethical business models. In detail, the literature analysis also provided a new conceptual framework and a set of research propositions to be developed in the future, both from a theoretical and empirical point of view. This study contributes to the academic debate identifying the main dimensions of the technological innovations useful to spread RI values and practices to achieve SDGs. Likewise, this SLR and bibliometric analysis suggest to managers the main directions achieve innovative business models, including ethical values to meet SDGs.

The following is a breakdown of the study’s structure. The next section introduces the Theoretical Background. Methodology, Data Analysis and Results, and Discussion of results are presented in the next sections. After that, theoretical and Managerial Implications, Policy Recommendations, Limitations of the Study, and Avenues for Future Research and Recommendations are discussed in subsections. The conclusion section ends this study.

## Theoretical background

### From corporate social responsibility to responsible innovation and ethical corporate behaviour

In the business management literature, the concept of business responsibility has been extended progressively from an individual company’s economic responsibility to make a profit for its shareholders to a wider moral and ethical responsibility towards society. It has been conceptualized differently, CSR (Carroll & Shabana, 2010) being one of the most common definitions and being boosted and replaced by “corporate sustainability”, which is currently considered a precondition for business survival (Jarmai et al., 2020). With the development of global supply chains, companies’ behaviours are being scrutinized for their impact on society and the environment (Phillips & Caldwell, 2005), extending their responsibility beyond their operations to supply chains, including raw material procurement, production, packaging, transport, and disposal after use. Companies are expected to assume ethical responsibility for not harming people or the environment. In contrast, the most advanced ones investigate strategies that drive the business through responsiveness to societal needs (Jarmai et al., 2020). In this change of perspective, innovation, generally conceived as the basis for business competitiveness, has been invested with the role of finding substitute solutions on the market that are more eco-friendly, sustainable, ethical, and socially desirable and thus combining the pursuit of competitiveness with the requirement to reduce harm to people and the natural environment (Adams et al., 2016).

The literature on sustainable innovation has identified several potential drivers for integrating sustainability into companies’ innovation strategies and practices. Traditionally based on innovation theory, these drivers have been classified into supply-side factors, demand-side factors, and the regulatory framework (Jarmai et al., 2020). Supply-side factors include technological and managerial capabilities, tangible and intangible assets, and knowledge and skills. Demand-side factors include the market demand and customers’ perception of the company. The regulatory framework consists of laws, regulations, and standards, such as the International Organization for Standardization (ISO), which is an important driver of the implementation of sustainability-oriented innovation in businesses. Fichter and Clausen (2021) proved how external factors (i.e., technological innovation, market demands, regulation and support mechanisms, and public opinion) are processed within a specific organization, mostly depending on internal structures and values as the company culture and the intrinsic motivation. Kumar et al., (2020, p. 804) argued: “that organizations need to develop capabilities to adapt to customer expectations and understand customer idiosyncrasies while constantly engaged in innovation and value creation processes”. According to Gil-Gomez et al. (2020), competitiveness and opportunities to improve productivity or reduce costs play a pertinent role in adopting business innovations. However, implementing a responsible business strategy may require a fundamental shift in mindset from simply adhering to laws and regulations to actively creating a positive impact on society and/or the environment. Engaging with sustainability and responsibility issues requires gathering and processing knowledge from external sources. Agreeing upon standards of ethical and responsible conduct in research and innovation, consulting with external ethics advisors, and staying up to date on the latest data security regulations require commitment, skills, and time. Hence, fashion companies have to strike a balance between economic, social, and environmental goals to develop a sustainable supply chain that satisfies all their stakeholders’ expectations (Arrigo, 2021).

### Legitimacy theory, responsible innovation, and business model innovation

The decision to allocate manufacturing activities to Asian countries, due to the wide availability of a trained, low-cost workforce, low taxation, and weak regulations, raises ethical and environmental issues that can undermine the image and reputation of focal companies. The dispersion of suppliers in geographically distant contexts, especially those of fast-fashion retailers, the strategies of which are cost-oriented, makes it hard for the management to control the negative effects deriving from low sustainable practices (Arrigo, 2020). Environmental and social sustainability, and related ethical issues, can represent a relevant hidden cost (Larsen & Lawson, 2013) that fashion companies should not disregard in their outsourcing strategies. Legitimacy theory suggests that to gain legitimacy and face market competitiveness, companies may adopt innovations to change their products, practices, or processes in response to social and institutional pressures regarding sustainability requirements. To address the increasing pressure exercised by consumers, policymakers, and stakeholders (Khan et al., 2021; Singh et al., 2021) and enhance their legitimacy, companies are trying to integrate sustainability and ethics into their business models. Likewise, the pressure on firms due to the global competition needs innovative and efficient processes to achieve legitimacy (Tsinopoulos et al., 2018). Hence, the relationship between open innovation and legitimacy theory becomes stronger and stronger. Indeed, open innovation initiatives promote the use of external sources in the business models advancing its technological and knowledge capabilities (Chesbrough, 2003). Under the lens of a global competition, legitimacy is also achieved through integrating innovations from external sources and sharing resources and innovation processes with all partners (West & Bogers, 2014). Innovation can radically change companies’ business, altering the organization of their activities with customers, suppliers, partners, and other stakeholders across the firm and industry boundaries. As a result, firms’ business model, or “the system of interdependent activities that transcends the focal firm and spans its boundaries”, is redesigned (Zott & Amit, 2010, p. 216).

Business model innovation (BMI) has been defined “as a business model new to the industry in which the focal firm competes; that is, without known precedent in that industry” (Snihur & Zott, 2013). The adoption of BMI implies the definition of new organizational exchanges and establishing connections with other partners. Specifically, scholars have highlighted that the adoption of the RI approach strongly depends on the entrepreneur, the managers, and the value system and facilitates alignment with the company’s mission and the organic nature of the company’s structure; these effects occur with the leadership and spread across the organization and its suppliers, consumers, financers, and so forth (Dicuonzo et al., 2020; Singh et al., 2019). Despite several innovations requiring effective appliances to the market to show their potential, the method with which firms adopt BMI is still unknown (Chesbrough, 2007; Foss & Saebi, 2017). These concepts are combined, focusing on the use of BMI as a leverage point to sustain organizations in achieving their sustainability goals (Bocken et al., 2015; Evans et al., 2017; Geissdoerfer et al., 2016).

### Stakeholder theory and responsible innovation

RI research focuses on how organizations and all stakeholders take joint responsibility for creating value in sustainable, ethical, and mutually desirable ways (Owen et al., 2013; von Schomberg, 2013). Beyond the creation of economic value, RI aims at “maximizing the economic and social benefits (or impact) of science and innovation” (Owen et al., 2013, p. 29), not only generating private value but contributing to value creation for society through innovative solutions that can range from the products and processes to innovative business models.

A significant dimension of RI concerns how innovation is governed, which is called responsible governance (Scherer & Voegtlin, 2020; von Schomberg, 2013). Responsible governance has the task of managing and controlling the desirable ends of innovation to ensure they are effective (Owen et al., 2013). It calls for the focus to be moved from the effects (or products) to the purposes of RI, prompting questions about the intentions and motivations behind adopting RI tools and the stakeholders who will benefit from the economic and social impacts produced by innovation. To achieve their responsible innovation goals, companies need to define “effective stakeholder governance, when the value created gets allocated sustainably and desirably to their set of intended stakeholders” (Bacq & Aguilera, 2022, p. 30).

According to Bacq and Aguilera (2022), corporate governance theories present two main limitations that can prevent the application of RI; they are mostly anchored to the economic value concept, aiming at its maximization, and overlook the value distribution among the multiple stakeholders who contributed to its generation. The adoption of RI in business organizations requires responsible governance based on three pillars: multi-stakeholder decision-making processes, redistribution of power and consensual procedure rights. This entails the establishment of mechanisms of interaction among multiple stakeholders through deliberative democratic processes concerning how value is created, for whom, and how it is distributed. The value created by the focal organization through innovation has to be delivered to all the contributing stakeholders (Bacq & Aguilera, 2022, p. 33). In this regard, Owen et al. (2013) “suggest a need for substantive processes of inclusive reflection and deliberative democracy”. The concept “deliberative engagement” refers to the organization of democratic and inclusive innovation processes by involving different stakeholders, including non-professionals; in this way, innovators can understand the social desirability of innovation and become responsive to societal values and needs (Brand & Blok, 2019). Stakeholder groups and community members are encouraged to participate early in the innovation process, deliberating over the many uncertainties that the innovation may bring, contributing to the realization of collective responsibility, and driving innovation in a direction that is ethically acceptable, societally desirable, and sustainable (Von Schomberg, 2013). This will increase the possibility of innovation adoption and, at the same time, the acceptance of innovation in society, guaranteeing more significant social benefits (Ribeiro et al., 2016).

### Open innovation and responsible innovation

The management literature has extensively discussed approaches to establishing an innovation culture within an organization, the pros and cons of involving external company actors, and the selection process of one idea over another. Well-known management approaches to decreasing uncertainty through utilizing information from company-external sources include open innovation (Chesbrough et al., 2006; Fichter, 2009; Gassmann & Enkel, 2004; von Hippel, 1986). According to open innovation theory, business development strongly depends on interaction and exchange with other businesses, generating new knowledge, which becomes a source of their competitive advantage (Del Giudice et al., 2013a, 2013b; Della Peruta & Del Giudice, 2013). This means that business development depends “on a company’s ability or inability to fragment and distribute the knowledge it requires to operate successfully in environments that are continuously modified by innovation, competition, and institutional change” (Della Peruta et al., 2018, p. 682). The widely accepted definition of open innovation is “a distributed innovation process based on purposively managed knowledge flows across organizational boundaries, using pecuniary and non-pecuniary mechanisms in line with the organization’s business model” (Chesbrough & Bogers, 2014, p. 17). Chesbrough et al., (2006, p. 174) explained that “Since knowledge flows more readily to closer entities (Jaffe et al., 1993), the organization and institutional embeddedness of geographical networks might be crucial in explaining the differences in the effectiveness of open innovation in different regions or nations”. These aspects are significant in the fashion industry, the value chain distributed among several partners and suppliers located in geographically, culturally, and institutionally distant contexts. Starting with the open innovation framework, RI extends the previously developed concepts related to knowledge sharing, stakeholder engagement, and deliberative engagement by including ethical and social responsibility in the innovation process (Sudolska et al., 2019). RI relies on the ability to anticipate events and introduce innovation capable of generating a competitive advantage and on the moral engagement of stakeholders and organizations through ECB, regardless of the consequences.

**Fig. 1:**
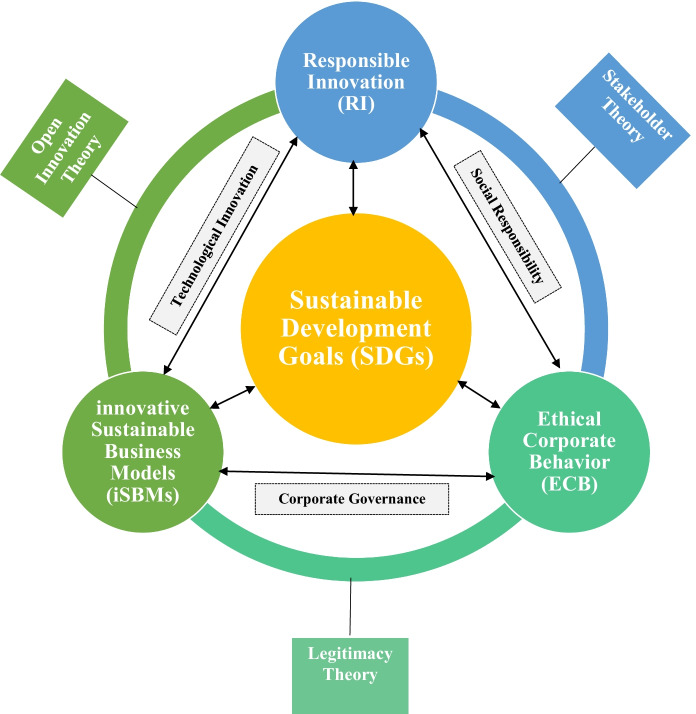
“Theoretical framework on RI and iSBMs through ECB towards SDGs”. Source: Authors’ elucidation

New societal challenges necessitate rethinking ethical, social, and environmental considerations beyond simple conformity to established norms. These call for moral innovation based on the individual virtues of entrepreneurs, top managers, and employees and their ability to produce ethical and socially responsible outputs once specified normative limitations had been considered. Brand and Blok (2019, pp. 10–11) “suggest that significant changes in corporate governance are required if RI goal is to be achieved structurally” (Fig. 1).

## Methodology

This study is based on an SLR using a replicable, scientific, and transparent process (Tranfield et al., 2003). The SLR analysis the role of theories supporting our theoretical framework on RI and BMIs through ECB which is a subject field of this study, to advance the body of literature (Paul & Criado, 2020). SLR, compared with other types of review, presents the following strengths: a) higher quality of the process and outcomes (Leonidou et al., 2017); b) bias minimization (Dada, 2018); c) greater validity due to the replicability of the steps followed by the authors (Wang & Chugh, 2014); d) a clear map of the research area investigated (Kauppi et al., 2018); and e) provision of the pillars on which authors can propose a new conceptual framework (Dada, 2018; Lim et al., 2022). This study has adopted a mixed quantitative and qualitative approach.

Specifically, we used bibliometric reviews to analyze a wide number of studies already published on the issues here investigated by using statistical tools to understand in-depth trends, methods, journals, countries, specific topics, theories (Paul & Criado, 2020). This study, it was made use of VOSviewer software version 1.6.15 for bibliometric network design and development widely used for conducting this kind of bibliometric review (Paul & Criado, 2020; Van Eck & Waltman, 2014). VOSviewer is open-source software that allows the mapping visualization of keywords and the construction of co-occurrence networks linked to subject areas. In addition, VOSviewer supports the text-mining feature, thus helping to gain a deeper and richer understanding of our bibliographic findings. To perform the descriptive bibliometric analysis (source type, document type, yearly trend, authors per document, most prolific and cited articles, organizations, and countries), we exported the publication meta-data to MS Excel 2019 (Microsoft, Rochester, NY). The bibliometric and network analysis, citation analysis, co-authorship analysis, and co-occurrence networks analysis were conducted using the VOSviewer program “version 1.6.15” (Van Eck & Waltman, 2014). A qualitative analysis was also conducted by analyzing the contents of all the selected articles included in our data collection to identify, investigate, and report patterns in the form of the topics proposed within our study.

This study analyzed 114 publications between 1990 and 2021 from the Scopus database and Google Scholar (GS). Scopus has been considered the biggest curated and reviewed abstract and index database for academics, governments, and business organizations (Elsevier, 2022).

**Fig. 2:**
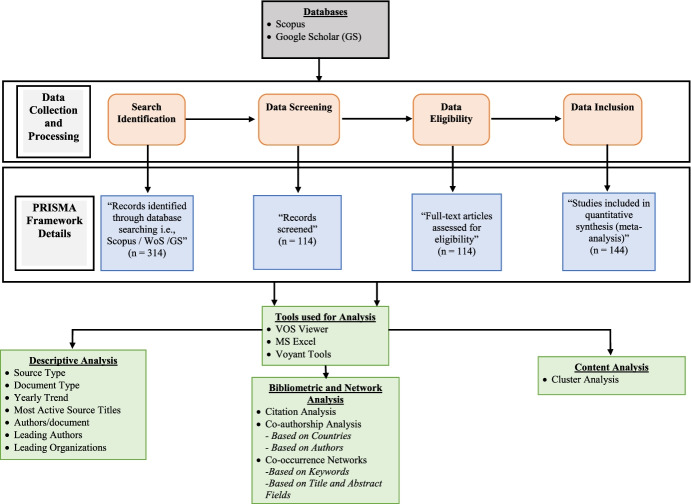
**“**Study’s research design and methodology”. Source: Author’s elucidation

It is wider than the ISI Web of Science (WoS). Therefore, we decided to integrate our database with a manual content search conducted on GS to increase the coverage of our research field. We selected all documents (i.e., conference papers, book chapters, articles, and books) (Bonilla et al., 2015) related to RI, BMI, CSR, and ECB, paying attention to those investigating these topics in the fashion industry, as well as the context, i.e., Asia. Following the protocol adopted in the previous SLR, our research design is structured in five main steps, as shown in Fig. 2. In the first phase, we launched several searches in Scopus through the truncated association of nine search string categories to obtain relevant articles:

- Group 1 – responsible innovation AND business models AND innovative processes
- Group 2 – corporate ethical behaviour AND innovative processes
- Group 3 – corporate ethical governance AND innovative processes
- Group 4 – corporate ethical governance AND responsible innovation
- Group 5 – responsible innovation AND knowledge management
- Group 6 – corporate ethical governance AND responsible innovation AND knowledge management AND SDGs
- Group 7 – corporate ethical governance AND Asia AND fashion industry
- Group 8 – technological innovation AND corporate ethical governance AND Asia AND fashion industry
- Group 9 – technological innovation AND corporate ethical governance AND Asia AND fashion industry AND innovative business models

The nine configurations aimed to bring in as many subject-related articles as possible and validate the similarities among the papers reviewed in the various study categories. Indeed, the current study focused on the position of RI in shaping the corporate behaviours of Asian fashion firms, leading them to adopt innovative and sustainable business models able to achieve the SDGs.

In the second research step, we conducted a content analysis of all the abstracts to highlight the correspondence with our research aims and exclude those that were distant from our issues.

In the third research step, assuming that the Scopus database might not capture all the papers relevant to our focused domain and analysis, we undertook a manual search of GS, utilizing the consistent factors and consulting the main journals known to publish articles on RI, CSR, and ECB, such as the *Journal of Cleaner Production*, *Journal of Business Research*, *Journal of Responsible Innovation*, *Academy of Management Perspectives* (Okoli and Schabram, 2010), *Journal of Business Research*, *International Journal of Information Management*, and *Business and Strategy and the Environment*, to avoid excluding any relevant reference for our research.

In the fourth step, all the abstracts were read after deleting redundant studies and duplications, eliminating articles that were not linked to our research purposes. The final list contained 114 articles.

In the fifth step, we launched the VOSviewer to visualize the conceptual network. This allowed us to understand the authors’ main keywords and their connections.

The sixth research step involved distinguishing individual authors, contrasting their findings, and separating them into parts to evaluate the selected articles; all the documents were viewed, and their essential features underlined. This approach allowed us to answer our RQs, evidence the existing gaps, design a new conceptual framework, and outline the propositions for future research on the issues proposed in this study.

## Data analysis and results

The sub-sections that follow provide a quantitative analysis of the selected studies. This was conducted through an in-depth bibliometric analysis of the final article data set centered on institutions, country of publication, authors’ contributions, journals, and a yearly number of publications (Howard et al., 2017) utilizing the bibliographic data collected from the Scopus database and GS.

### Bibliometric aspects of the selected articles

#### Keyword analysis

**Fig. 3:**
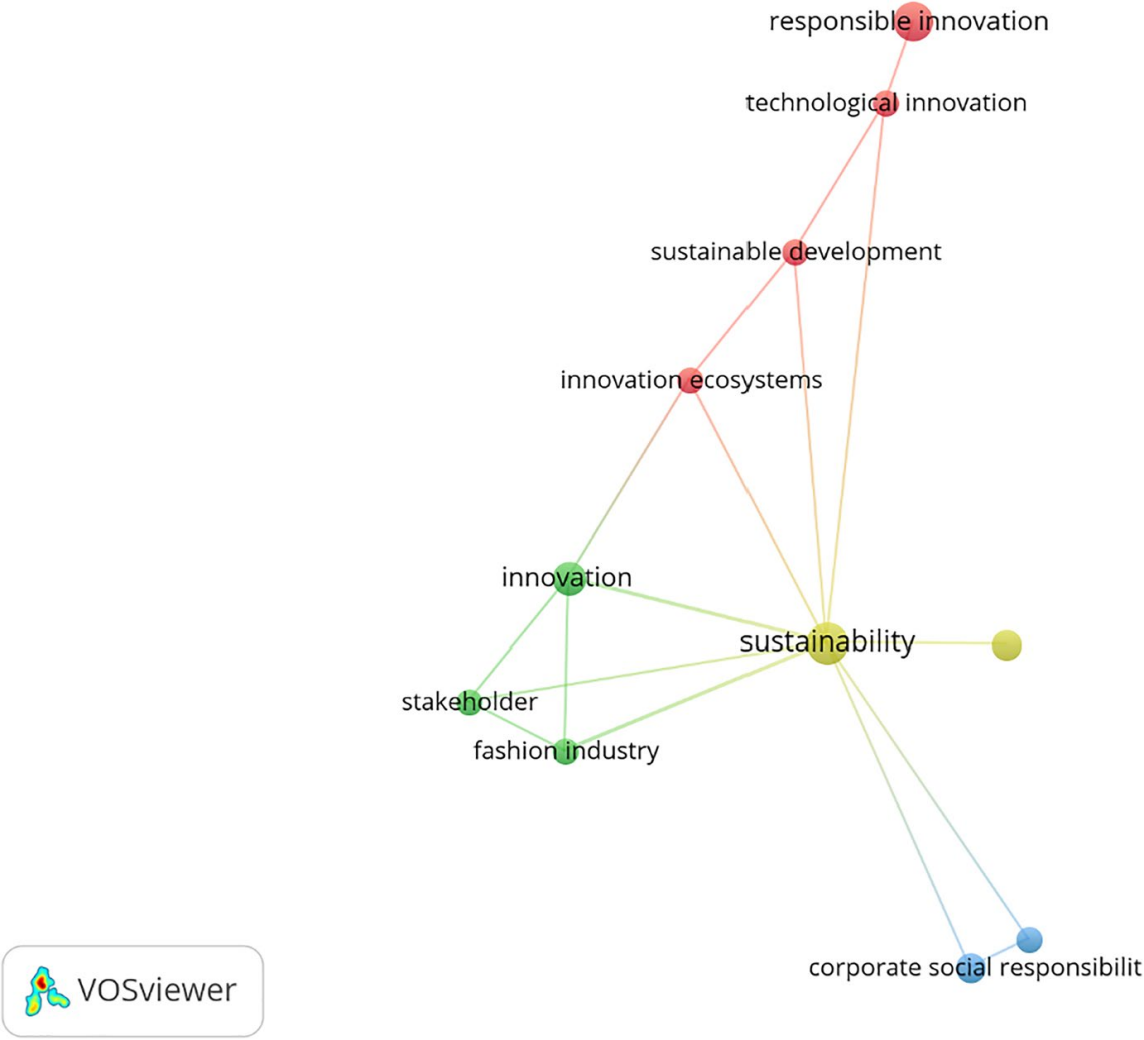
“Network visualization map of the author keywords”. Source: Authors’ elucidation using VOSviewer

Using the VOSviewer 1.6.15 text-mining tool (Van Eck & Waltman, 2014), we obtained a holistic view of the keywords used by the authors of the articles constituting the final data set. This advanced method has been validated in recent bibliometric articles (Marzi et al., 2017). The text-mining method helps to create a map and is based on the distance between different keywords and their relationships. The more significant the distance between two or more keywords, the more significant the association between them. The publications’ co-occurrences were analyzed to judge the words’ interconnectivity (Van Eck & Waltman, 2014). The analysis of the keywords is based on “Author Keywords”, and these keywords occur at least twice in the database. This study relied on manual selection to ensure data reliability, resulting in 37 keywords out of a total of 177 being considered appropriate for the analysis. This filtered out the keywords (e.g., study, content analysis, literature, and so forth) that could not explain anything independently. Figure 3 presents the author and keyword network visualization map and the most frequently used keywords in selected papers. In the figure, the word size is based solely on the inclusion criteria of the chosen articles. Figure 3 highlights the main keywords as “sustainability”, “responsible innovation”, “innovation”, and “corporate social responsibility”, and these appear at the center of the map. They were used during the period of study as a constant in the data collection (Amatulli et al., 2018; Brand & Blok, 2019; Cao et al., 2020; Chou, 2018; del Mar Ramos-González et al., 2021; Di Vaio et al., 2020; Hasan, 2016, 2018; Hemphill & White III, 2018; Herrera, 2016; Hillestad et al., 2010; Imaz & Eizagirre, 2020; Koberg & Longoni, 2019; Li et al., 2020a, 2020b; Medik & Stettina, 2014; Nathan, 2015; Nayak et al., 2019; Popescu et al., 2016; Ranabahu, 2020; Scherer & Voegtlin, 2020; Steen et al., 2021; Sudolska et al., 2019; Thomas & da Silva, 2019; Yadlapalli et al., 2018). In several papers reviewed, RI, BMI, and CSR interfaces appeared.

The conceptual map was created to show the association between keywords of selected data using bibliometric analysis (Van Eck & Waltman, 2010). Figure 3 depicts the keywords and co-occurrence or co-word estimation and some well-known RI, BMI, CSR, and ECB themes from the literature. For example, as the color matching indicates, the terms “responsible innovation”, “technological innovation”, and “sustainable development” have a relationship; it is important to note ex multis. This co-occurrence index shows the strength of the relationship between RI and sustainable development by propagating RI, improving ECB, and contributing to changing business models to achieve sustainability goals. The top keywords used by several researchers in the past are presented in Table 1.

**Table 1:** Top Keywords

Author Keywords	Frequency	Percent
Sustainability	7	3.95
Responsible Innovation	6	3.39
Innovation	4	2.26
Corporate Social Responsibility	3	1.69
Social Sustainability	3	1.69
Corporate Governance	2	1.13
Fashion Industry	2	1.13
Innovation Ecosystems	2	1.13
Stakeholder	2	1.13
Sustainable Business Models	2	1.13
Sustainable Development	2	1.13
Technological Innovation	2	1.13

Total Key words = 177

Source: Authors’ elucidation using VOSviewer & MS Excel

#### Documents and source types

In the sample, 80.70% of the 114 articles contained the most diverse forms of documents. These were closely followed by papers from conferences (6.14%). Table 2 provides a complete synopsis of the various type of papers.

**Table 2:** Document Type

Document Type	Frequency	% (N = 114)
Conference Paper	07	6.14
Article	92	80.70
Book Chapter	04	3.51
Review	06	5.26
Editorial	0	0.00
Article in Press	05	4.39
Conference Review	0	0.00
Note	0	0.00
Book	00	0.00
Letter	0	0.00
Short Survey	0	0.00
Erratum	0	0.00
**Total**	**163**	**100.00**

Source: Authors’ elucidation using VOSviewer & MS Excel

Journals (93.86%) were the most numerous sources of the documents. In Table 3, a detailed overview of the sources is presented.

**Table 3:** Source Type

Source Type	Frequency	% (N = 114)
Journals	107	93.86
Conference Proceedings	07	6.14
Book Series	0	0.00
Books	0	0.00
Trade Publications	0	0.00
**Total**	**114**	**100.00**

Source: Authors’ elucidation using VOSviewer & MS Excel

#### Years of publication − Evolution of published studies

**Fig. 4:**
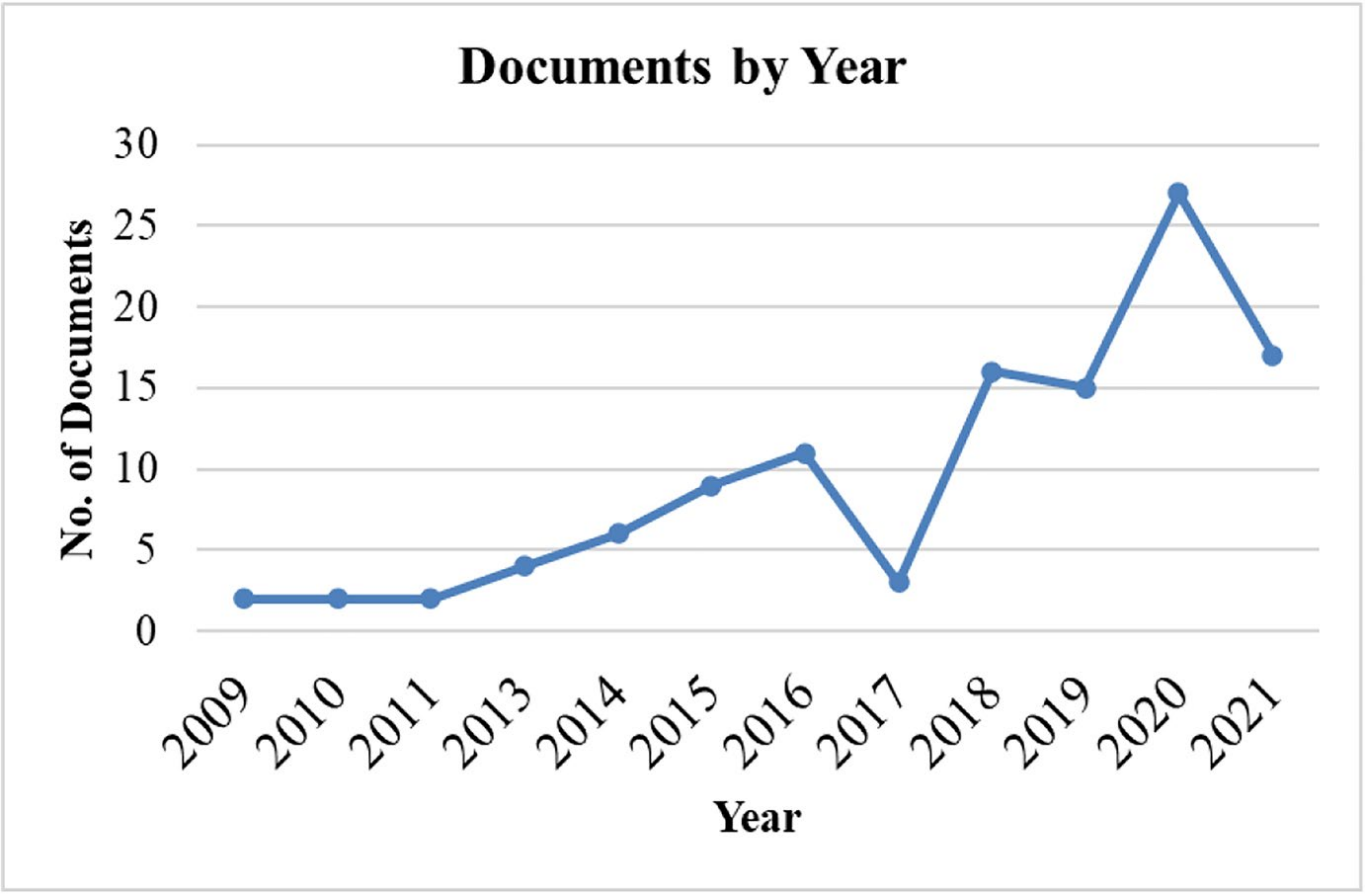
“Documents by Year”. Source: Authors’ elucidation using VOSviewer & MS Excel

Figure 4 shows the development of publications from 2009 to 2021 in our selected area of research. A gradual increase in RI, BMI, CSR, and ECB publications have emerged. Conversely, the lowest number of publications was observed in the 2009–2011 period, followed by growth between 2012 and 2021 except in 2017. This demonstrates researchers’ growing interest in RI, BMI, CSR, and ECB. Table 4 shows a complete list of publications between 2020 and 2021, with the maximum interest in RI and the fashion industry.

**Table 4:** Year of Publications

Year	Frequency	% (N = 114)	Cumulative Percent
2009	2	1.75	1.75
2010	2	1.75	3.51
2011	2	1.75	5.26
2013	4	3.51	8.77
2014	6	5.26	14.04
2015	9	7.89	21.93
2016	11	9.65	31.58
2017	3	2.63	34.21
2018	16	14.04	48.25
2019	15	13.16	61.40
2020	27	23.68	85.09
2021	17	14.91	100.00
**Total**	**114**	**100.00**	

Source: Authors’ elucidation using VOSviewer & MS Excel

#### Most active source titles

The foremost and most active journals that have published papers linked to RI, BMI, CSR, and ECB are summarized in Table 5. The *Journal of Cleaner Production*, *Journal of Business Research*, and *Academy of Management Perspectives* are examples. These source titles contain important papers on RI, BMI, CSR, and ECB. They have examined the effect of RI in shaping the corporate behaviours of Asian fashion firms, leading them to adopt innovative SBMs (Amatulli et al., 2018; Herrera, 2016; Koberg & Longoni, 2019; Li et al., 2020b; Nayak et al., 2019; Ranabahu, 2020).

**Table 5:** Most Active Source Title

Source Title	No. of Documents	%
“Journal of Cleaner Production”	4	22.22
“Journal of Business Research”	2	11.11
“Academy of Management Perspectives”	1	5.56
“Advances in Business Marketing and Purchasing”	1	5.56
“Business Sustainability in Asia: Compliance, Performance, and Integrated Reporting and Assurance”	1	5.56
“Journal Of Business Ethics”	1	5.56
“Knowledge Management Research and Practice”	1	5.56
“Leading Responsibly in the Asian Century”	1	5.56
“Public Policy and Administration”	1	5.56
“Sustainability (Switzerland)”	1	5.56

Total source titles = 18

Source: Authors’ elucidation using VOSviewer & MS Excel

#### Geographical distribution of publications: The most influential countries

The list of the top 15 countries publishing articles is presented in Table 6. Australia (3.51%) is the leading country, followed by Spain (2.63%). This finding indicates that the original central pillar for RI, BMI, CSR, and ECB studies was Australia and Spain. Interestingly, Bangladesh (1.75%) and Malaysia (1.75%) are ranked third in Asia. Despite the governments of both Bangladesh and Australia taking appropriate measures, Australia has recorded a high growth rate and encouraged studies on RI, BMI, CSR, and ECB (Hasan, 2016, 2018). In the context of Spain, many studies have focused on RI, BMI, CSR, and ECB to ascertain the causes of evolving digitalization in different countries. Portugal, Switzerland, and Turkey appear at the bottom of the table, contributing 0.88% of the total publications. Another critical point is that no studies from world-leading countries with industry-based economies, such as South Korea, China, Canada, and so on, are available for RI, BMI, CSR, and ECB. In these countries, cultural and business contexts with the present requirements and research focused on RI, BMI, CSR, and ECB were more often substantiated.

**Table 6:** Top 15 Countries contributed to the publications

Country	Frequency	% (N = 114)
Australia	4	3.51
Spain	3	2.63
Bangladesh	2	1.75
France	2	1.75
Malaysia	2	1.75
United States	2	1.75
Denmark	1	0.88
Germany	1	0.88
Hong Kong	1	0.88
Italy	1	0.88
Lithuania	1	0.88
Nepal	1	0.88
Portugal	1	0.88
Switzerland	1	0.88
Turkey	1	0.88

Total countries = 20

Source: Authors’ elucidation using VOSviewer & MS Excel

#### Authorship

Table 7 shows the publishing trend with the total number of authors per paper: three authors (28.95%), followed by two (27.19%), one (23.68%), and four (14.91%), and so on. It can confidently be stated that published papers with more than one author seem to have higher quality and attract more academic attention.

**Table 7:** Number of Author(s) per document

Author Count	Frequency	% (N = 114)
1	27	23.68
2	31	27.19
3	33	28.95
4	17	14.91
5	5	4.39
6	1	0.88
10	1	0.88
**Total**	**114**	**100.00**

Source: Authors’ elucidation using VOSviewer & MS Excel

Regarding the most productive authors in the field of RI, BMI, CSR, and ECB, Rahim (Australia) and Scherer (Switzerland) top the table, with one document each with the highest number of citations, followed by one document by Di Vaio (Italy) and all the other authors with one document with eight citations, as presented in Table 8. Interestingly, the articles all have a combination of authors of different genders, and they are all from developed countries.

**Table 8:** Most Productive Authors with one document and a minimum of eight citations

Author’s Name	No. of Documents	Percentage (%)	Citations
Rahim M.M	1	1.67	21
Scherer A.G	1	1.67	17
Voegtlin C	1	1.67	17
Costa J	1	1.67	11
Matias J.C.O	1	1.67	11
Di Vaio A	1	1.67	8
Escobar O	1	1.67	8
Hassan R	1	1.67	8
Palladino R	1	1.67	8

Total Authors = 60

Source: Authors’ elucidation using VOSviewer & MS Excel

**Fig. 5:**
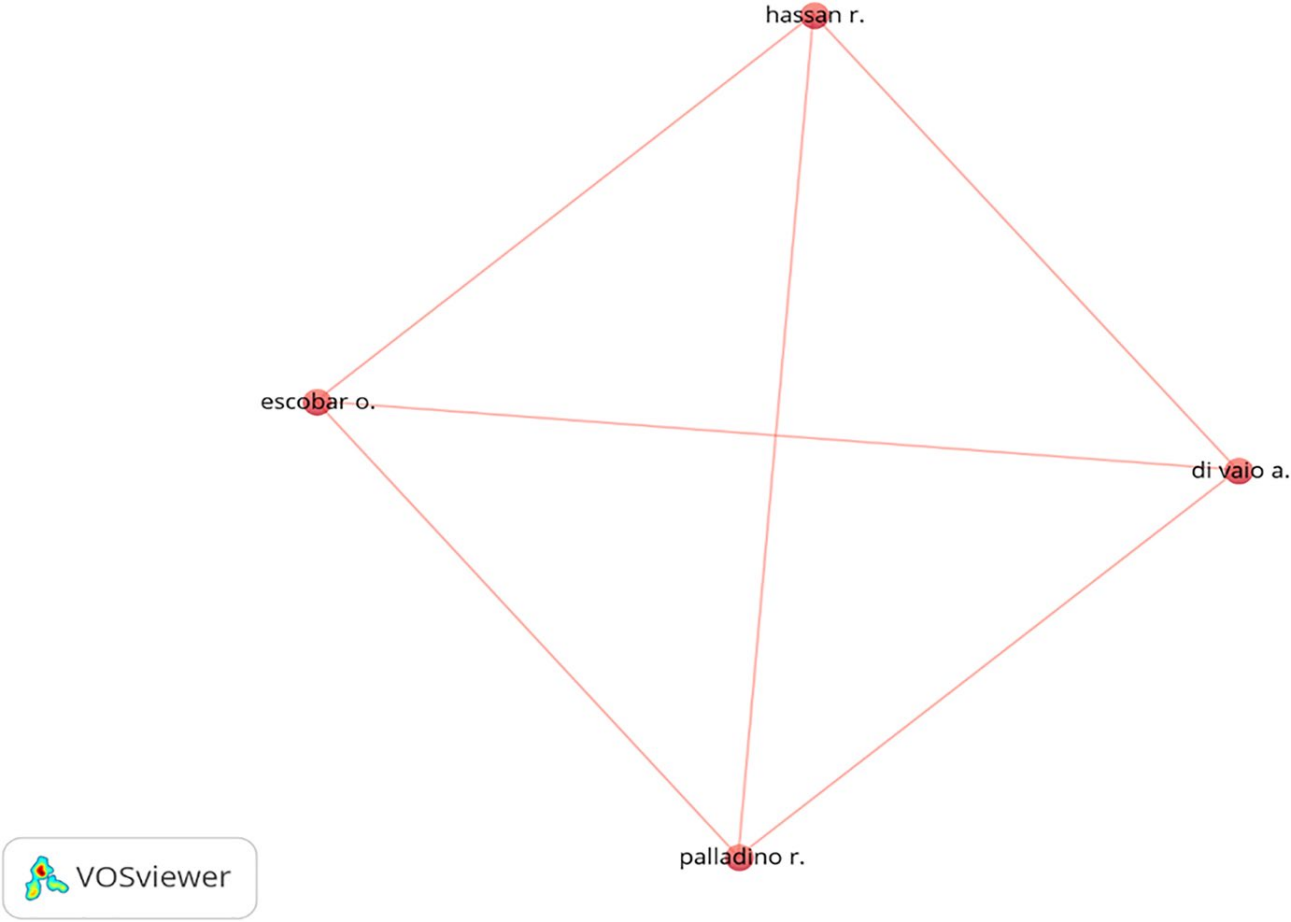
“Network visualization map of the co-authorship”. *Source: Authors’ elucidation using VOSviewer.* “Unit of analysis = Authors”. “Counting method: Fractional counting”. “Minimum number of documents of an author = 1”. “Minimum number of citations of an author = 5”

**Fig. 6:**
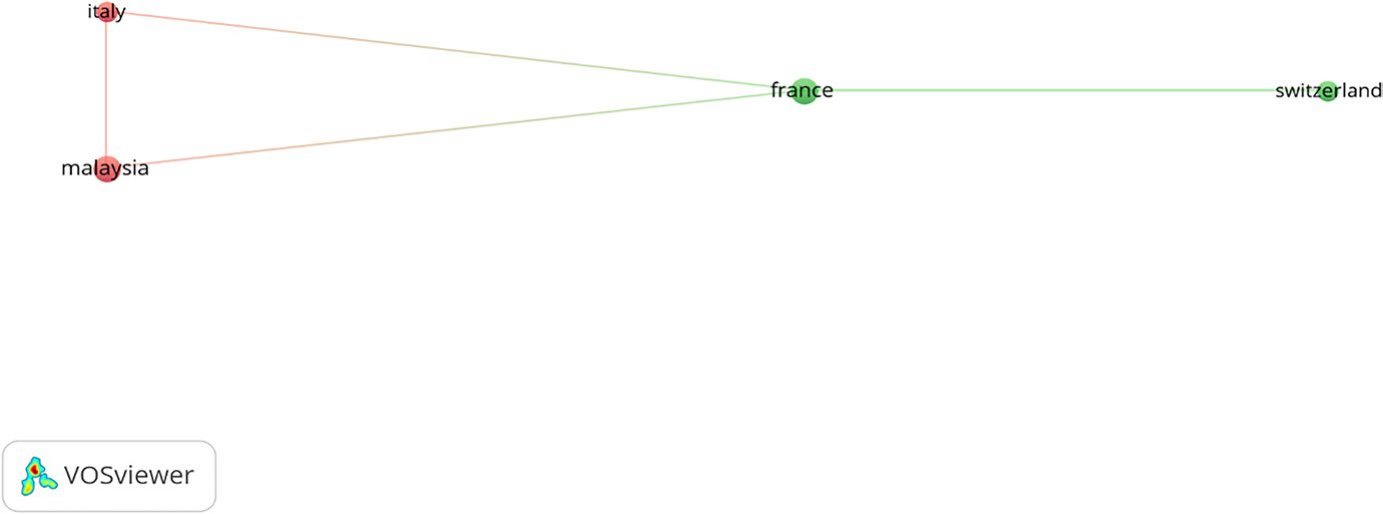
“Network visualization map of the co-authorship”. *Source: Authors’ elucidation using VOSviewer.* “Unit of analysis = Countries”. “Counting method: Fractional counting”. Minimum number of documents of a country = 1. Minimum number of citations of a country = 1

Cooperation between scholars is necessary to improve any field; therefore, further cross-country cooperation is needed (Turner and Baker, 2020). Figures 5 and 6 present the co-authorship and country trends among scholars using authors and countries as the unit of analysis. France, Italy, Switzerland, and Malaysia are the leading nations in joint efforts. This shows an interesting network that covers several continents. Di Vaio, Escobar, Hassan, and Palladino have collaborated most with researchers from various countries. Cultural relations, geopolitical position, and language are the factors that decide and shape co-authorship preferences (Schubert & Schubert, 2020). This study highlights that geopolitical similarity and language are essential in co-authorship. There is, however, a broader output of Australian research papers and a more remarkable willingness on the part of its academics to cooperate with their colleagues in other countries.

#### Most influential institutions

Table 9 displays the top institutions within the RI, BMI, CSR, and ECB literature with a minimum of one publication with the highest numbers of citations. Equal numbers of papers have been contributed by the RMIT University, Australia, and other RMIT schools (2). However, in terms of citations, the most popular article is from the University of South Australia, Adelaide, and its School of Law. The article from the University of South Australia thus tops the list of the most famous articles.

**Table 9:** Most influential institutions with a minimum of one publication with four citations

Institution	Frequency	% (N = 39)	Citations
“School of Accounting, College of Business, RMIT University, Melbourne, Australia”	2	5.13	6
“School of Business It and Logistics, College of Business, RMIT University, Melbourne, Australia”	2	5.13	6
“Audencia Business School, France”	1	2.56	17
“Degeit-Department of Economics, Management, Industrial Engineering and Tourism, University of Aveiro, Campus Universitário De Santiago, Aveiro, 3810–193, Portugal”	1	2.56	11
“Department of Law, University of Naples, Parthenope”, Via G. Parisi, No. 13, Naples, 80,132, Italy”	1	2.56	8
“EM Normandie Business School, Métis Lab, 64 Rue Du Ranelagh, Paris, 75,016, France”	1	2.56	8
“Faculty of Business, University of Wollongong, Wollongong, NSW, Australia”	1	2.56	6
“Govcopp-Research Unit on Governance, Competitiveness and Public Policies, Campus Universitário De Santiago, Aveiro, 3810–193, Portugal”	1	2.56	11
“Hadimkoy Yolu, Buyukcekmece, Istanbul, Turkey	1	2.56	7
Istiklal Mah. 2. Cad. No:21/B, Memenen, Izmir, D:10, Turkey”	1	2.56	7
“Othman Yeop Abdullah Graduate School of Business (OYAGSB), Universiti Utara Malaysia, UUM Sintok, Kedah Darul Aman 06,010, Malaysia”	1	2.56	8
“School of Law, University of South Australia, Adelaide, Australia”	1	2.56	21
“University of Massachusetts, Dartmouth, United States”	1	2.56	4
“University of Zurich, Switzerland”	1	2.56	17

Source: Authors’ elucidation using VOSviewer & MS Excel

#### Citation analysis

According to Bornmann et al. (2008), the influence of a piece of research is the degree to which other scientists have found it helpful. The citation metrics from 2009 to 2021 are based on 114 records, as presented in Table 10. The cumulative number of citations over 14 years is 1653, resulting in 14.50 citations per paper and 137.75 citations per year. Citations signify the quality of the various other publications that authors used regarding research findings, other research ideas, and so on. Hence, it can be concluded that the number of citations serves as a determining factor of the research’s impact (Bornmann & Daniel, 2007).

**Table 10:** Citations Metrics

Metrics	Data
Publication years	2009—2021
Citation years	12 (2009—2021)
Papers	114
Citations	1653
Citations/year	137.75
Citations/paper	14.50
Citations/author	27.55
Papers/author	1.9
Authors/paper	0.53

Source: Authors’ elucidation using VOSviewer & MS Excel

Table 11 indicates the most cited authors. Islam and Deegan (2010) can be observed at the top of the list with their widely cited article “Media pressures and corporate disclosure of social responsibility performance information: A study of two global clothing and sports retail companies”, identifying RI and CSR evolution, applications, and emerging research fields, that is, corporate disclosure and social responsibility with regard to performance.

**Table 11:** Highly cited articles—Most Influential Papers

No	Authors	Title	Year	Cites	Cites per Year
1	Islam M.A., Deegan C	“Media pressures and corporate disclosure of social responsibility performance information: A study of two global clothing and sports retail companies”	2010	170	15.45
2	Huq F.A., Stevenson M., Zorzini M	“Social sustainability in developing country suppliers: An exploratory study in the readymade garments industry of Bangladesh”	2014	143	20.43
3	Van Bommel H.W.M	“A conceptual framework for analyzing sustainability strategies in industrial supply networks from an innovation perspective”	2011	112	11.20
4	Huq F.A., Chowdhury I.N., Klassen R.D	“Social management capabilities of multinational buying firms and their emerging market suppliers: An exploratory study of the clothing industry”	2016	109	21.80
5	Koberg E., Longoni A	“A systematic review of sustainable supply chain management in global supply chains”	2019	100	50.00
6	Li Y., Zhao X., Shi D., Li X	“Governance of sustainable supply chains in the fast fashion industry	2014	80	11.43
7	Jia F., Zuluaga-Cardona L., Bailey A., Rueda X	Sustainable supply chain management in developing countries: An analysis of the literature”	2018	68	22.67
8	Hillestad T., Xie C., Haugland S.A	“Innovative corporate social responsibility: The founder's role in creating a trustworthy corporate brand through "green innovation"	2010	61	5.55
9	Perry P., Wood S., Fernie J	“Corporate Social Responsibility in Garment Sourcing Networks: Factory Management Perspectives on Ethical Trade in Sri Lanka”	2015	60	10.00
10	Winter S., Lasch R	“Environmental and social criteria in supplier evaluation – Lessons from the fashion and apparel industry”	2016	53	10.60
11	Hastig G.M., Sodhi M.S	“Blockchain for Supply Chain Traceability: Business Requirements and Critical Success Factors”	2020	43	43.00
12	Yadlapalli A., Rahman S., Gunasekaran A	“Socially responsible governance mechanisms for manufacturing firms in apparel supply chains”	2018	39	13.00
13	Schrempf-Stirling J., Palazzo G	“Upstream Corporate Social Responsibility: The Evolution from Contract Responsibility to Full Producer Responsibility”	2016	36	7.20
14	Kennedy A.-M., Kapitan S., Bajaj N., Bakonyi A., Sands S	“Uncovering wicked problem’s system structure: seeing the forest for the trees”	2017	33	8.25
15	Amatulli C., De Angelis M., Korschun D., Romani S	“Consumers’ perceptions of luxury brands’ CSR initiatives: An investigation of the role of status and conspicuous consumption”	2018	32	10.67
16	Herrera M.E.B	“Innovation for impact: Business innovation for inclusive growth”	2016	29	5.80
17	Haque M.Z., Azmat F	“Corporate social responsibility, economic globalization and developing countries: A case study of the readymade garments industry in Bangladesh”	2015	28	4.67
18	Kucharska W., Kowalczyk R	“How to achieve sustainability? —Employee's point of view on company's culture and CSR practice”	2019	26	13.00
19	Mezzadri A	“Backshoring, local sweatshop regimes and CSR in India”	2014	24	3.43
20	Huq F.A., Stevenson M	“Implementing Socially Sustainable Practices in Challenging Institutional Contexts: Building Theory from Seven Developing Country Supplier Cases”	2020	23	23.00

Source: Authors’ elucidation using VOSviewer & MS Excel

#### Textual exploration

**Fig. 7:**
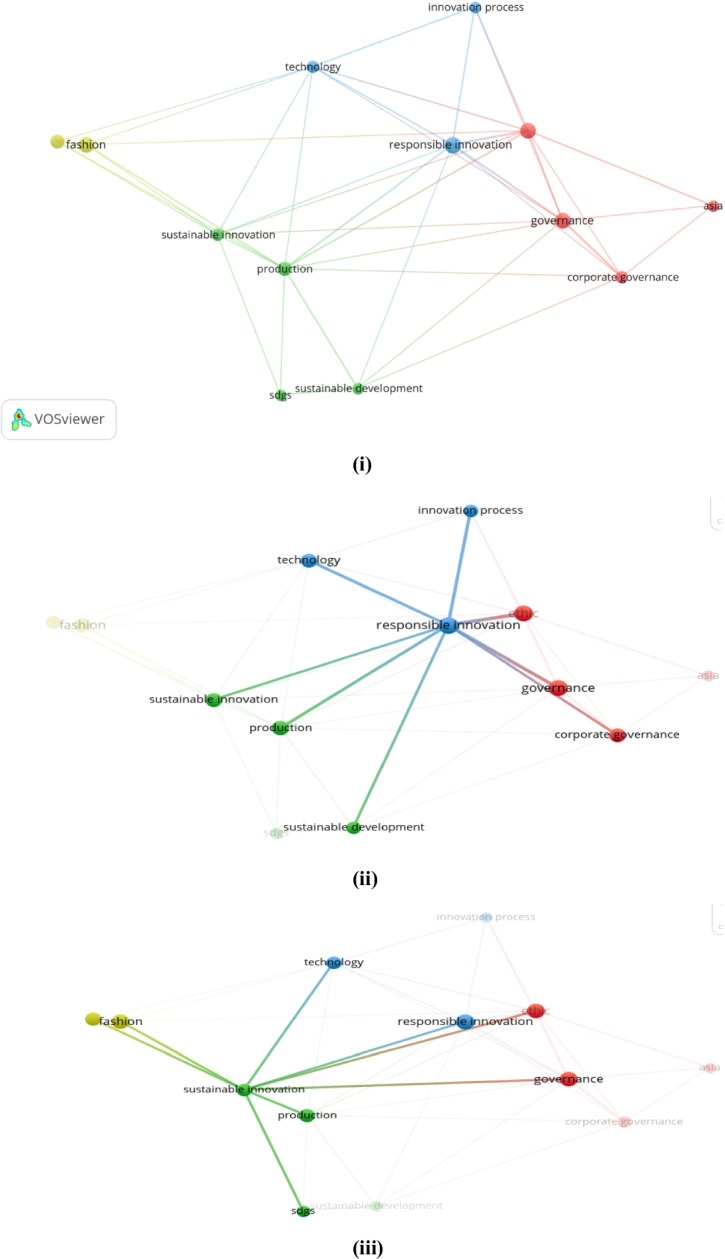
**(i, ii, & iii):** “VOSviewer visualization of a term co-occurrence network based on title and abstract fields (Binary Counting)”. Source: Authors’ elucidation using VOSviewer

Keywords can be discovered, assessed, and offered systematically through VOSviewer. A map was generated based on bibliographic facts to show a co-word network. The strength of the association was utilized to standardize the concepts of the interaction of keywords (Van Eck & Waltman, 2007, p. 2). Each phrase was graphically placed on the map through the visualization of similarities (VoS) method (Van Eck & Waltman, 2014). The VOSviewer algorithm offers multiple options for detecting different clusters based on resolution. This study zeroed in on 21 keywords in this case; the relative solid associations of co-occurrence were measured with other keywords. Colors (green, blue, yellow, and red) subsequently differentiated four distinct clusters. Figures 7 (i, ii, & iii) and 8 (i & ii) contain the graphical representation of the co-occurrence or co-words of keywords.

**Fig. 8:**
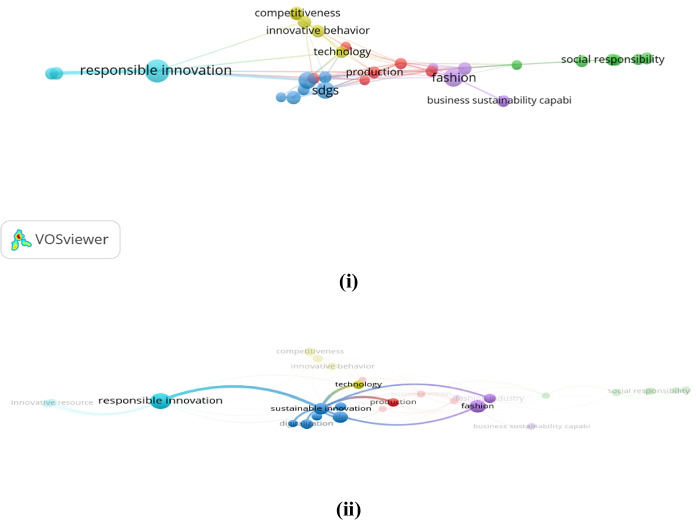
**(i & ii):** “VOSviewer visualization of a term co-occurrence network based on title and abstract fields (Full Counting)”. *Source: Authors’ elucidation using VOSviewer*

Figure 7 (i, ii, & iii) clarifies the framework of knowledge or concepts of the previous studies in general (Di Vaio et al., 2020). The software produces circles of different colors and sizes to explore the terms. The circle size signifies a specific term; if it is large, it appears with a high frequency in abstracts and publication titles (Van Eck & Waltman, 2014). The observed distance between the circles is valid; the bigger the distance between two circles, the better the relationship. This relationship is solely established through the number of occurrences in the titles and abstracts of the articles that the words present collectively (Van Eck & Waltman, 2014). The occurrence of case nine or more times was the criterion for inclusion, and 21 words were finally included. The VOSviewer described four different clusters and attributed four different colors to them depending on their thematic community.

Figures 7 (i, ii, & iii) and 8 (i & ii) show the title and abstract fields’ co-occurrence network. The role and potential of RI in shaping the corporate behaviours of Asian fashion firms, leading them to adopt innovatively and SBMs able to achieve the SDGs, are presented in these figures. Therefore, the government’s goal and well-structured fashion industries should focus on RI, BMI, CSR, and ECB with regard to the SDGs to achieve better business performance.

Through the schematization of the subtitles and the short explanation of each article’s intent, the classification of the 114 articles revealed that most scholars had analyzed generally the consequences of the relationship between CSR and sustainability (Amatulli et al., 2018; Brand & Blok, 2019; Cao et al., 2020; Chou, 2018; del Mar Ramos-González et al., 2021; Di Vaio et al., 2020; Hasan, 2016, 2018; Hemphill & White III, 2018; Herrera, 2016; Hillestad et al., 2010; Imaz & Eizagirre, 2020; Koberg & Longoni, 2019; Li et al., 2020a; Medik & Stettina, 2014; Nathan, 2015; Nayak et al., 2019; Popescu et al., 2016; Ranabahu, 2020; Scherer & Voegtlin, 2020; Steen et al., 2021; Sudolska et al., 2019; Thomas & da Silva, 2019; Yadlapalli et al., 2018).

#### Content of the selected articles

Our study provides evidence that the activation of innovation processes strictly depends on internal knowledge, leadership, and corporate culture, but it is also customer, normative, and technology-driven. The role that corporate culture plays in innovation is widely acknowledged, but the relationship between RI and corporate culture is mainly theoretical (Cao et al., 2020; Chou, 2018; Costa & Matias, 2020; Imaz & Eizagirre, 2020; Nathan, 2015; Ranabahu, 2020; Scherer & Voegtlin, 2020; Steen et al., 2021; Sudolska et al., 2019). To achieve entrepreneurial goals, “corporate culture” is defined as the “rules and norms of behaviour based on material and spiritual values, cultural, ethical, and social demands of employees”. Bag et al. (2018) suggested that corporate culture should be formed through multiple-step processes that start from the definition of business priorities and encompass the formation of employees’ behaviour, the implementation of effective, innovative activities, and the involvement of all stakeholders in the sphere of innovative entrepreneurship. Stakeholder engagement especially facilitates business model innovation and co-creation by enabling production and knowledge collection (Akbar & Ahsan, 2020; Brand & Blok, 2019; Huq et al., 2014, 2016; Lathabhavan, 2021; Li et al., 2014, 2020b; Nayak et al., 2019; Venkatesh et al., 2021).

Our study for RQ1 reveals that RI is gaining traction among academic scholars and policymakers to address some ethical business challenges, particularly regarding social and environmental sustainability. In recent years, a growing literature has investigated the antecedents and consequences of adopting RI for business sustainability. In the fashion industry, RI is particularly heartfelt, and most studies have considered to be it strictly linked to the concept of CSR (Akbar & Ahsan, 2020; Huq & Stevenson, 2020; Huq et al., 2016; Kennedy et al., 2017; Lee et al., 2018; Nayak et al., 2019; Thanh Liem et al., 2019; Pal & Gander, 2018; Popescu et al., 2016; Rahim, 2017; Winter & Lasch, 2016).

In the fashion industry, the spread of RI appears to be strictly linked to global fashion brands’ adoption of sustainable strategies. They are progressively adopting sustainable practices and technologies to comply with stricter regulations and gain legitimacy among consumers and stakeholders. However, most global fashion brands are consumers of textiles and garments produced and processed by developing and under-developed nations (Li et al., 2020a, b). Thus, achieving sustainability in the fashion industry includes promoting CSR throughout the whole chain of suppliers. Scholars have used the concept of sustainable supply chain management (SSCM) in various studies (Bag et al., 2018; Del Pilar Quiroz Galvan et al., 2021; Fontana et al., 2021; Fung et al., 2021; Handfield et al., 2020; Hastig & Sodhi, 2020; Hemphill & White, 2018; Huq & Stevenson, 2020; Huq et al., 2014, 2016; Jia et al., 2018; Koberg & Longoni, 2019; Li et al., 2014; Nayak et al., 2019; Pal & Gander, 2018; Schrempf-Stirling & Palazzo, 2016; Winter & Lasch, 2016; Yadlapalli et al., 2018; Yang et al., 2019; Zhou et al., 2020) to refer to the sustainable practices that many global fashion brands are striving to develop along their supply chain. Despite several sustainable practices having been adopted in leading Asian fashion manufacturing countries, such as Vietnam or Bangladesh, some studies have proved that the concept of a sustainable supply chain is new to several actors (Huq et al., 2014; Nayak et al., 2019; Winter & Lasch, 2016). A frequent obstacle to the adoption of SSCs in the fashion industry is the misalignment of corporate ethics, cultural values, and socio-economic conditions among Western and Asian countries. Employees in different institutional contexts, such as the United States and China, may have varying moral standards based on their work country; hence, their business sustainability commitment can vary dramatically (Lee et al., 2018). Some research has investigated the practices, relationships, processes, and skills that can help “buyers” and “suppliers” to respond to stakeholder pressures, address regulatory gaps, and improve their social performance (Huq et al., 2016). “Audits”, “monitoring”, “open discussion”, and “trust” between parties are the possible enablers (Huq et al., 2014). Supposing that sustainable and innovative leadership of the focal company is essential to drive the change in terms of RI (Winter & Lasch, 2016), knowledge sharing, participation, and collaboration with suppliers and stakeholders appear to be equally important for the fashion industry to build sustainable and innovative business models in developing countries. As widely discussed in the literature, innovation is a process that is based primarily on internal knowledge production, management, and sharing among all stakeholders (Bakhov et al., 2020; Beltramino et al., 2020; Borowski, 2021; Del Río Castro et al., 2021; Mai, 2011; Venkatesh et al., 2021; Zhou et al., 2020).

In this regard, a relevant role is played by technological innovation (RQ2) and new technologies (Costa & Matias, 2020; Di Vaio et al., 2020). The role of technological innovation in improving efficiency and boosting sustainability, allowing companies to function, work, and develop in an ever-changing environment, has been acknowledged (Cao et al., 2020; Costa & Matias, 2020; Del Río Castro et al., 2021; Nathan, 2015), but it remains primarily unexplored in the fashion industry. There is an ongoing debate on the opportunities and consequences of digital development and automation in the fashion industry. While efficiency for businesses will increase, some key social issues regarding employment should also be considered. In developing countries like Bangladesh, replacing workers with advanced digital technologies will improve efficiency and flexibility, reduce the lead time, increase precision, and reduce the environmental impact through decreased waste and less polluting emissions. New technologies indeed allow business models to switch from a “low-tech labour-intensive approach” to a “high-tech knowledge-intensive approach”, but their implementation strongly depends on the socio-economic conditions (Rinaldi, 2020). The development of highly technological innovations requires relevant capital investments, which are usually implemented in developed countries. Besides, the level of knowledge transfer is high, so their adoption throughout the whole fashion supply chain could be profitable in long-term and trust-based partnerships.

According to previous research (Di Vaio et al., 2020), digital technologies, particularly artificial intelligence (RQ3) can serve as a vehicle for achieving the SDGs, enabling the recognition of the cultural shifts businesses need to make to attain the long-term goals. Theoretically, digital technologies can support the fashion industry in achieving the SDGs in several ways. First, they could ensure the traceability of the whole value chain (raw materials to sale), significantly enhancing the level of business transparency regarding their products’ social and environmental sustainability. It can also involve consumers in the design process through customization, engaging them in production and recycling processes, decreasing the environmental impact, and favoring SDG12. This study highlights that the key enablers of SDGs’ implementation can be found more in partnership and innovation (horizontal enablers) than in business principles and value (vertical enablers) (Imaz & Eizagirre, 2020), while Sinkovics et al. (2020) proposed a responsibility matrix to map firms’ activities and their contributions to the achievement of the SDGs.

## Discussion of results

**Fig. 9:**
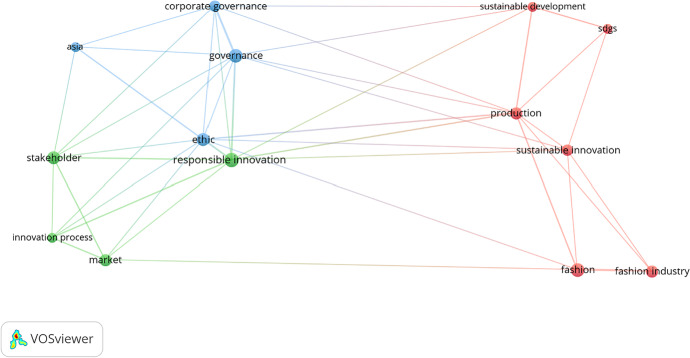
“VOSviewer visualization of a term co-occurrence network based on abstract fields (Binary Counting)”. Source: Authors’ elucidation using VOSviewer

**Fig. 10:**
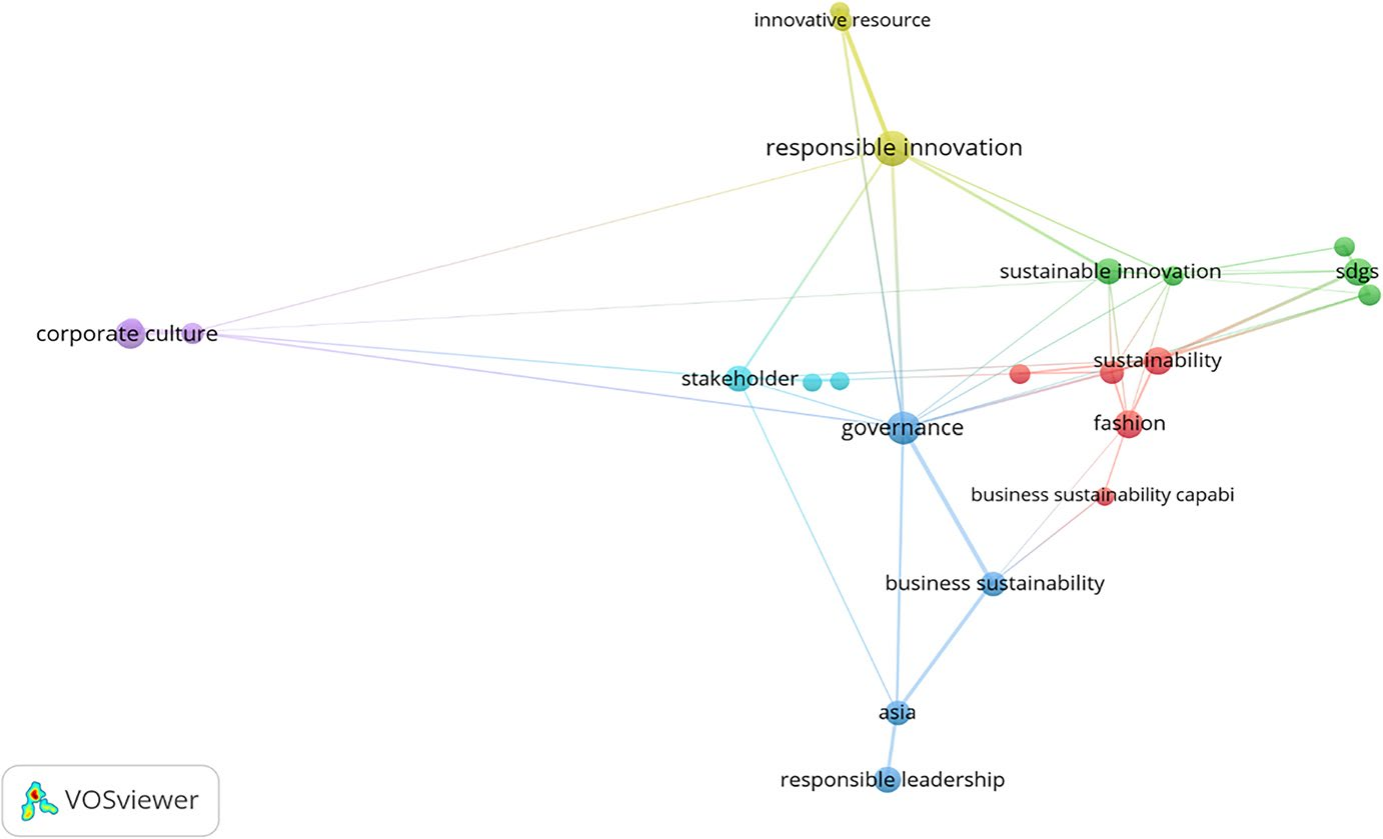
“VOSviewer visualization of a term co-occurrence network based on abstract fields (Full Counting)”. Source: Authors’ elucidation using VOSviewer

Our results highlight the growing interest of scholars in SSCM in the fashion industry. The results suggest that RI, BMI, CSR, and ECB are linked to the development of IBMs for the SDGs (Chou, 2018; Nathan, 2015; Steen et al., 2021; Sudolska et al., 2019), and new technologies can assess the efficiency of their process and increase the sustainability of the fashion industry (Figs. 9 & 10).

Regarding RQ1, “What is the role of responsible innovation in enabling ECB in the fashion industry?”, there is an urgent need to extend the focus of research on the fashion industry to the application of RI principles to SSCM and to the mechanisms that can help to transform a reactive approach into a proactive approach. A recent study (Bhandari et al., 2022) conducted on the fashion industry revealed that “insufficient commitment from top management” represents the main barrier to sustainable sourcing in the apparel and fashion luxury industry, followed by the poor awareness of “sustainable sourcing”, “inadequate infrastructure”, and “social sustainability”. Green innovation is considered essential for gaining a competitive advantage in a sustainable supply chain to achieve the SDGs (Zhou et al., 2020). Technological innovation has been widely acknowledged as the main driver of sustainable business model development. However, recent studies (Uniyal et al., 2021) conducted on Asian countries have empirically evidenced that “‘Governance and Management’, is the topmost factor for the adoption of sustainable consumption and production in value chains”.

In relation to RQ2, companies’ practical application of the RI framework requires the institutionalization of a deliberative process with suppliers and stakeholders on the purposes, strategies, and possible impacts that the focal company intends to pursue through its innovations. This would require the redesigning of the current business models, characterized by the exclusive localization of manufacturing activities in Asian countries due to the wide availability of a trained, low-cost workforce, low taxation, and weak regulations, to build new models based on the sharing of strategies, knowledge, and values between the focal company and its suppliers. Recent studies have highlighted the existence of barriers to spreading RI values in developing countries, especially the cultural and socio-economic differences between Western and Asian countries, which are also reflected in their corporate codes and values (Jia et al., 2018; Lee et al., 2018). Possible enablers have been identified in establishing collaboration and partnerships with suppliers and sub-suppliers in which common values, procedures, and standards across the supply network should align interests and behaviours (Bag et al., 2018; Huq & Stevenson, 2020). These would allow firms to achieve multiple benefits, such as “lower development costs, lower project cycle completion time, improvement in design for re-manufacturability, low downtime, low supply risks, reduction of greenhouse gas emissions and, ultimately, sustainable development” (Bag et al., 2018). Indeed, some recent experiences have shown that fashion brands have started to localize in Asian countries’ business headquarters to manage and monitor business activities closely in these countries. This opens the space for new business models that could change fashion brands’ competitive strategies, from their cost-efficiency orientation to sustainable and ethical value propositions.

Thus, from our perspective, it is becoming essential to identify the RI components that can influence cost efficiency and produce positive impacts on fashion companies’ value proposition, increasing their competitiveness (Figs. 9 & 10). However, boosting sustainable supply chain development through green innovation is a complex network activity in which a large number of partners are embedded, and there is a need to transfer or share knowledge in an equal and reasonable exchange process. Embeddedness and knowledge sharing exert a significant partial mediating impact on green innovation in the sustainable supply chain; knowledge sharing plays a key role in achieving green innovation.

Concerning RQ3, business organizations have realized that the expertise and talents of their employees, that is, knowledge and human capital, help them to achieve success. This study asserts that implementing strategies to mitigate RI and BMI is essential for sustainable development and improving the evidence base to enhance future technology explorations. It provides evidence that, despite the various unique opportunities RI application offers to the fashion industry, its exploration is still in its infancy and further research, examination, and understanding are necessary.

Adopting the RI innovation framework entails the responsibility of established institutions, structures, and procedures to facilitate innovations that “do not harm” and “do good”. As a result, the dimensions reflect the fundamental characteristics of what we have characterized as responsible innovation, such as sustainability, innovation process feasibility, acceptability, and its consequences.

When those stakeholders who care about social welfare and sustainable development are involved in corporate decision-making procedures, the stakeholder approach is exceptionally powerful in contributing to RI (e.g., as an advisory panel to the board of directors or when socially responsible investors can hold large blocks of shares). However, this is frequently dependent on the characteristics of stakeholders. In terms of efficiency, “doing things right” is essential from a normative and strategic stakeholder standpoint. The availability of a governance mechanism that can convert all of these factors into social or technological innovations that aid sustainable development and thus promote the development and diffusion of responsible innovations is dependent on the knowledge and competence of various stakeholders, their willingness to participate, and the availability of a governance mechanism that can transform all of these factors into social or technological innovations that contribute to sustainable development. Hence, our results highlight RI is a pillar in managing iSBMs. However, RI can be more effective than "sustainable innovation" alone, but under conditions that RI is incorporated into the issues of corporate culture and ethics of the business organizations involved in sustainable supply chain management (SSCM)(Bag et al., 2018). Likewise, our results find that the main obstacle to adopting SSCs in the fashion industry is the mismatch of corporate ethics, cultural values, and socio-economic conditions between the Asian context and the other mainlands (Lee et al., 2018). Thus, to reduce this gap, according to Winter and Lasch (2016), the knowledge sharing in the SSCM, which involves Asian countries, needs sustainable and innovative leadership to design and make sustainable and innovative business models in developing countries.

### A holistic comparison of our results with those of past studies

Even though RI is a comparatively new topic in the area of management studies (Blok & Lemmens, 2015; Owen et al., 2013; Von Schomberg, 2013), the major findings reveal that numerous studies have focused on its function in facilitating ECB (Cao et al., 2020; Čeičyte and Petraite, 2018; Chou, 2018; Costa & Matias, 2020; Imaz & Eizagirre, 2020; Nathan, 2015; Ranabahu, 2020; Scherer & Voegtlin, 2020; Steen et al., 2021; Sudolska et al., 2019), but most of the debate remains theoretical.

The CSR approach still dominates the discussion in the fashion industry, with several contributions highlighting the importance of spreading sustainable values and practices among leading manufacturing countries such like Bangladesh and Vietnam. Recent contributions have highlighted that the main drivers for the adoption of sustainable business models and innovation in the Asian fashion industry are the innovative leadership of top clothing brands and their needs to gain legitimacy with their customers and comply with stricter regulations (Del Pilar Quiroz Galvan et al., 2021; Fontana et al., 2021; Fung et al., 2021; Handfield et al., 2020; Hastig & Sodhi, 2020; Huq & Stevenson, 2020; Li et al., 2020a, 2020b; Yang et al., 2019; Zhou et al., 2020).

There is still a dearth of discussions about technological innovation in building innovative business models in the light of the SDGs, despite the growing research on BMI in the last decade (Baden-Fuller and Haefliger, 2013; Nosratabadi et al., 2019; Scheyvens et al., 2016; Teece, 2010; Zott and Amit, 2017, 2010). Despite the advanced technologies, BMI still requires some definitions for its successful introduction into the market, fashion companies still seem to be showing some reluctance to execute new business models with innovative technologies, and the academic research in this area remains scant (Boons & Lüdeke-Freund, 2013; Foss & Saebi, 2017; Schaltegger et al., 2016). Thus, the link between BMI or sustainability and technological innovation, that is iSBMs, and the role of new technologies in the fashion industry remains underexplored in details recent studies have focused on the support that technology can give to the traceability of the fashion supply chain, enhancing its image and reputation but empirical evidence is still missing on these issues (Cao et al., 2020; Costa & Matias, 2020; Del Río Castro et al., 2021; Nathan, 2015).

Based on a comprehensive review of these new studies, it is worth noting that research on advanced technologies in the development of BMI from the point of view of the SDGs is not yet unavailable in the academic literature, and they have not been thoroughly investigated. The growing attention that scholars are paying to the relevance of knowledge sharing among top clothing brand and their suppliers or customers and the active stakeholder participation required to promote RI in the fashion industry suggests an extension of studies in the coming years on the role of new technologies in the adoption of an RI approach in the fashion industry. These past studies have permitted authors to acknowledge the significance of new technologies in the development of BMI to meet sustainability goals. The expansion and efficacy of new technologies in the fashion industry aiming to achieve the SDGs have received little attention, and in-depth research into future trends in technological innovation to make the fashion industry more ethical and sustainable has yet to be published.

#### Theoretical implications of the study

The current research has practical and theoretical ramifications. It integrates the present literature by evidencing the critical business benefits of the RI and ECB fields’ comparatively underexplored areas. These could therefore be investigated further, particularly in developing countries. There is also insufficient literature on RI and the role of technological innovation in enabling Asian fashion companies to meet sustainability standards and achieve sustainability goals. This study suggests that to be incorporated into the corporate strategies and practices, RI requires the development of collaborations. Stakeholders need to be involved continuously in decision-making processes beyond mere consultation (Brand & Blok, 2019) and create what has been called “mutually beneficial interaction”. This is important in fashion businesses, the supply chain developed with several suppliers. Technological innovation plays a crucial role in breaking down barriers, facilitating traceability and transparency, and allowing knowledge and corporate values to flow and be shared.

Several contributions (Blok & Lemmens, 2015; Owen et al., 2013; Von Schomberg, 2013) have highlighted the significance of RI and ECB from a theoretical perspective. This study defines and presents previously new tensions in RI and CSR activities that challenge fashion firms’ capacity to provide the whole supply chain with RI support and meet the SDGs**.** It offers an understanding of the contribution that advanced technological innovations could make to spreading RI values and practices to achieve the SDGs.

#### Managerial implications of the study

Our research findings may give managers and practitioners valuable guidance. This study aids managers by advising them on how to design strategies for implementing responsible innovation practices in manufacturing plants. It reveals that RI alters Asian fashion companies’ corporate practices to improve their environmental and social performance, especially through global fashion brands’ innovation leadership. According to the conclusions of this study, managers should focus on adopting creative and sustainable business models that can satisfy the SDGs by implementing advanced technologies that can ensure the traceability of products alongside the whole fashion supply chain. This research also offers guidance for the fashion sector to build ethical innovation standards based on market needs. It suggests that engagement in RI requires the establishment of stable forms of collaboration and partnership, in which stakeholders interact continuously in decision-making processes. Open, clear, and transparent processes are the ingredients of trust-building and meaningful collaboration (Jarmai & Vogel-Pöschl, 2020). Technology can support this process, creating virtual spaces that enable knowledge sharing and collaboration. This study suggests that organizations should innovate their business by incorporating ethical values to meet sustainability goals and satisfy customers’ emerging needs, especially in the fashion industry, where the value chain is shared among suppliers that could have significantly different corporate values.

#### Policy recommendation

The emergence of an RI strategy is influenced by internal capabilities and resources and consumers’ awareness of environmental issues, legislation, innovation, and competitive dynamics (Lenidou et al., 2020). Practitioners, policymakers, regulators, and researchers have to focus more on RI, BMI, more specifically iSBMs, and ECB in the fashion industry. Asian policymakers should improve the institutional environment by changing the environmental and social regulations—mainly regarding workforce and human rights—to enable the adoption of RI, BMI, and CSR values and practices to achieve the SDGs. This approach will facilitate the avoidance of the potential gap between stakeholders’ views, regulatory requirements, and business practices. In addition, policymakers should adopt tailored rather than one-size-fits-all solutions to improve the business environment through the definition of common standards and when considering the possible levers that could help to accomplish the best combination and favor the creation of RI.

#### Limitations of the study

This study has several limitations. Firstly, the information on Scopus and GS changes regularly, leading to variations in the number of articles and citations (Valenzuela-Fernandez et al., 2019). One of the limitations of Scopus is that when authors or journals submit articles, thy are listed in Scopus only when they upload articles. Therefore, the accuracy of the data collected from the Scopus database and GS on a specific day is doubtful. Second, scientific mapping and profiling are quantitative approaches: they help to generate reports, offer a broad picture of a topic, and enable a “deep dive” into it. This study also has research limitations regarding the keyword co-occurrence analysis (co-word analysis). Certain types of publications in bibliometric records may be understated. The value of the co-word assessment depends solely on the indexing methods, and the authors had no control over this (Zupic and Čater, 2015). Therefore, a systematic review is an idiosyncratic approach that incorporates quantitative and qualitative processes and is suggested for upcoming investigations. This study’s primary limitation is that this analysis of 12 years of research on RI, BMI, CSR, and ECB is restricted to papers published in related journals. Finally, the study’s choice of keywords was based on literature research and the meaning of RI. There is the possibility of the existence of other related keywords.

#### Avenues for future research and recommendations

This study provides recommendations for authors, journal editors, and reviewers on how RI, BMI, more specifically iSBMs, and ECB add value to the fashion industry’s activities, improving the awareness and role of RI through new technologies to create long-term value. Based on a comprehensive literature review, the literature synthesis of present study revealed that the field is under-explored, especially concerning the role that those technological innovations play as key drivers to meeting the SDGs. Thus, to complement and expand our understanding, there is a need for further study. Future research could investigate this phenomenon through empirical analysis.

Future research questions to be addressed should concern the technologies adopted by companies to ensure effective deliberative engagement and knowledge sharing among focal companies, suppliers, and consumers. Scholars should try to understand how stakeholders with dissimilar values, or stakeholders who oppose the innovation, may be involved in the innovation process and how their inclusion could affect the development of BMI. Further RQs that need to be answered are how to transform a traditional supply chain into an ethical supply chain, whether ethics could be transferred through knowledge sharing, and which kind of collaborations can enhance this exchange. Future researchers may conduct a survey study to assess whether and how ethics have been incorporated into corporate values and strategies in Asian fashion firms. Other studies should investigate the impact of technologies on the cost function of fashion companies, allowing them to gain “ethical profits”. As a result of its limitations, the analysis’s conclusions should be applied cautiously in various contexts. Future researchers could conduct a survey study with respondents in other industries and investigate the RI, BMI, and ECB in which the firms intend to invest and the most important challenges they face in making this transition.

**Fig. 11:**
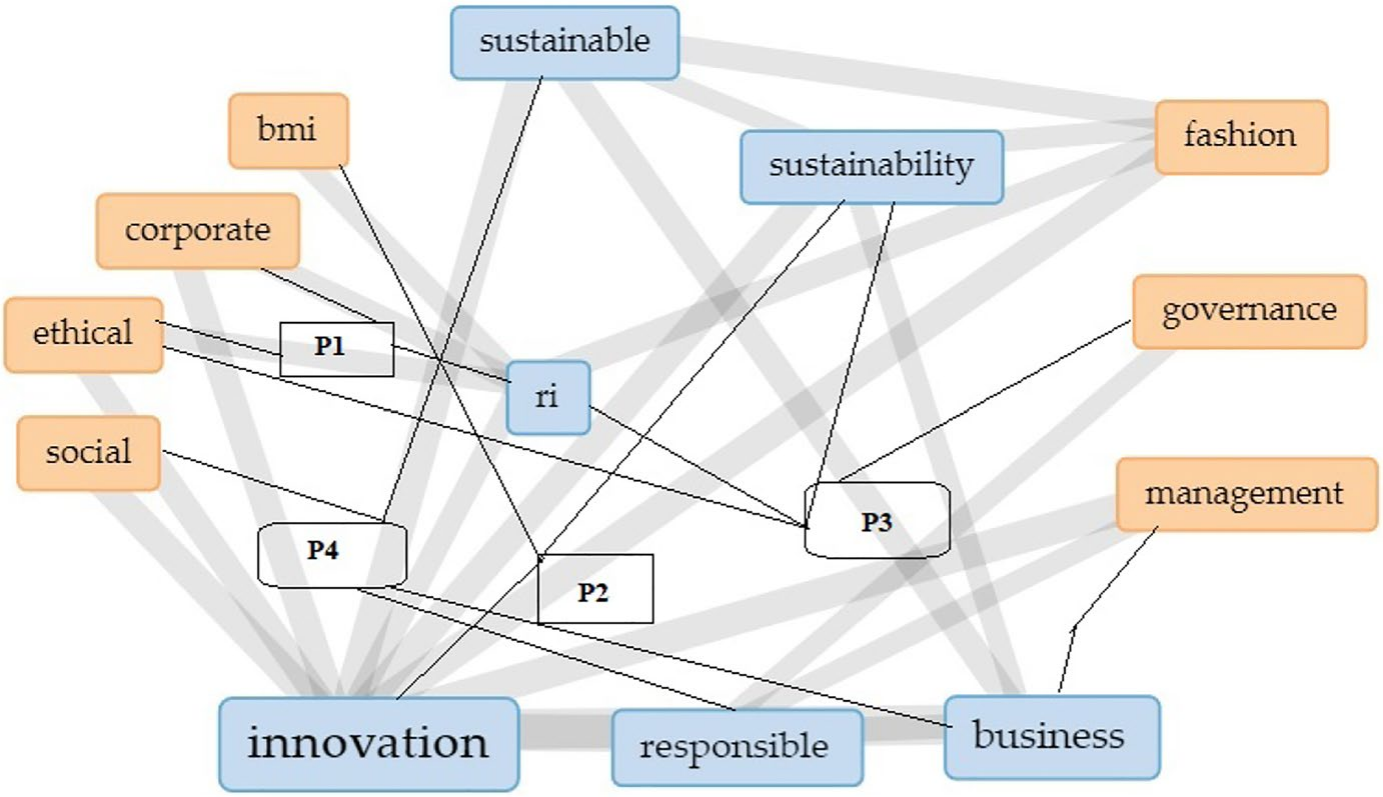
Conceptual framework for Responsible Innovation and Ethical Corporate Behavior. Source: Authors’ elucidation using Voyant Tools

As a result of the analysis, three RQs are discussed. According to Martens et al. (2013), the aim of the framework is content analysis, which encompasses the universe definition and sampling, the coding and interpretation of the results. To understand better the role of RI in enabling ECB in the fashion industry and spreading sustainability and ethical values throughout the whole supply chain, we presented a conceptual model linking the key components, variables, and interactions. This study contributes to the development of a conceptual framework for understanding the role of RI in enabling ECB in the fashion industry. These propositions are based on the study’s content analysis using Voyant Tools and the conceptual framework shown in Fig. 11. These graphs depict the direct and indirect relationships of the key variables used in this article. Furthermore, the width of the connecting lines indicates how strongly these variables are related. We also speculate on the direction (positive/negative) of the associations between the key variables to enrich the future research agenda (Lim et al., 2022), which should be explored and confirmed in future studies.

We developed the following group of propositions in response to the studies reviewed that dealt with RI and ECB issues. In the light of RQ1 (RI and ECB), we formed the following group of propositions.

The literature reviewed mainly discussed the relevance of the innovation processes, aiming to understand the appropriate ways in which business organizations can manage innovation from a responsibility perspective (Brand & Blok, 2019; Cao et al., 2020; Chou, 2018; Costa & Matias, 2020; Hartley et al., 2017; Imaz & Eizagirre, 2020; Nathan, 2015; Ranabahu, 2020; Scherer & Voegtlin, 2020; Steen et al., 2021; Sudolska et al., 2019). On the contrary, few studies have explored the role of RI in ECB as a function in facilitating ECB and linked it to concepts of CSR. Therefore, we put forward the following proposition:

##### Proposition P1

The relationship between RI and ECB is moderated by the corporate ethics, and cultural values represented.

Scholars have highlighted the need to manage and measure RI and BMI (Cao et al., 2020; Costa & Matias, 2020; Del Río Castro et al., 2021; Nathan, 2015). However, the role of new technologies in the fashion industry remains underexplored (Del Río Castro et al., 2021). Furthermore, such studies have neglected to examine the impacts of RI and ECB on BMI. Hence, it would be appropriate to focus future studies on RI to comprehend how stakeholders with divergent values, or stakeholders who are opposed to the innovation, may be involved in the innovation process and how their inclusion may affect the development of BMI. This drives our next proposition:

##### Proposition P2

New technologies influence the link between BMI or sustainability and technological innovation in the fashion industry.

Based on studies that have dealt with RI and ECB and in the context of RQ2, we developed the following group of propositions (technological innovation and ethics and responsibility).

Several scholars have pointed out the importance of technological innovation in helping to transform traditional business models into more sustainable ones and “advanced technologies” to improve the traceability and transparency of value chains to achieve a circular and sustainable economy (Caprani, 2016; Di Vaio et al., 2020; Vacchi et al., 2021). However, if this radical change is not accompanied by ethics and responsibility in governance and management, technological innovation may be ineffective (Blok & Lemmens, 2015; Von Schomberg, 2013). This means that RI is moderated by endogenous variables, which must be managed to achieve the SDGs. Nevertheless, it would be appropriate to develop a research focus on how technological innovation can be mediated through ethics and responsibility in governance and management by integrating them into companies as standard elements to ensure RI aspects. This leads to our next proposition:

##### Proposition P3

Technological innovation can be mediated by ethics and responsibility in governance and management through which companies operate and help to define RI.

Finally, based on studies that have investigated how technological innovation allows the adoption of innovative business models for the SDGs, in the context of RQ3, we developed the following proposition regarding technological innovation and SDGs. It remains unclear how companies use BMI despite several innovations that necessitate effective appliances to demonstrate their potential (Chesbrough, 2007; Foss & Saebi, 2017). Academics have recently developed tools and processes to aid organizations in developing business models. Breaking down barriers, facilitating traceability and transparency, and allowing knowledge and corporate values to flow and be shared are all aided by technological innovation. The development of long-term business models is largely credited to technological advancement. Thus, our next proposition is as follows:

##### Proposition P4

Technological innovation influences companies to adopt innovative business models for the SDGs.

A survey-based approach could be applied to answer the suggested research propositions on a future research agenda. Future studies could also use the model for some organizations with RI and analyse the impact on RI (by analysing BMI); explore the RI models proposed by the authors and the impact on ECB to meet the SDGs; analyze the influence or effect of the organizational culture on RI in greater depth, and analyze the governance aspects. Other aspects might emerge from the gap shown in this study. Future research could investigate this phenomenon through an empirical analysis. It needs to reach beyond organizational boundaries and offer an understanding of the meaning of RI, BMI, CSR, and ECB and their effects on society.

## Conclusion

The exponential increase in RI, BMI, and ECB in the fashion industry literature prompted this paper. Theory-based research on the role of RI, BMI, and ECB in the fashion industry is increasing, but empirical evidence is still lacking, despite both practitioners’ and scholars’ involvement. The study reveals the importance of determining how technological innovation may help Asian fashion companies to overcome the trade-off between profit and social responsibility. This also finds confirmation in the ASEAN Economic Community Blueprint (2025), which incentives the adoption of digital technology among Asian micro, small, and medium enterprises “as leverage to enhance trade and investments, provide an e-based business platform, promote good governance, and facilitate the use of green technology”.

This study provides evidence that technology is essential to ensure traceability and transparency, which are vital for building trusting relationships among companies and their stakeholders, including consumers, suppliers, and governments. Besides, technology enables the creation of virtual spaces, allowing knowledge-sharing exploitation and the active participation of stakeholders in the deliberative processes on which RI is grounded.

The urge to change the focus of research on technological innovation allows Asian fashion companies to adopt innovative business models focusing on the SDGs (explicitly or not), social capital, and innovation among people, stakeholders, and the organizational workforce, which help to translate information into creativity. The research underlines the potential disparities in the literature on the subject. This is, to the best of our knowledge, the first study to conduct a bibliometric and systematic review of RI, BMI, CSR, and ECB. The article intends to examine the specific opportunities and challenges that fashion companies face, recognizing that RI is in its adolescence stage and needs to be studied and examined further and understood better.
